# The potential role of nitrate, a nitric oxide donor, in the prevention and treatment of diabetic osteoporosis

**DOI:** 10.3389/fendo.2025.1480838

**Published:** 2025-06-12

**Authors:** Sajad Jeddi, Khosrow Kashfi, Asghar Ghasemi

**Affiliations:** ^1^ Endocrine Physiology Research Center, Research Institute for Endocrine Sciences, Shahid Beheshti University of Medical Sciences, Tehran, Iran; ^2^ Department of Molecular, Cellular and Biomedical Sciences, Sophie Davis School of Biomedical Education, City University of New York School of Medicine, New York, NY, United States

**Keywords:** diabetoporosis, fracture risk, nitric oxide, nitrate, osteoporosis

## Abstract

Approximately 28% of individuals with diabetes have osteoporosis. Diabetoporosis, which refers to the diabetes-related decrease in bone quality and quantity, increases the risk of osteoporotic fractures by 600-700% in individuals with type 1 diabetes (T1D) and by 38-70% in those with type 2 diabetes (T2D) compared to non-diabetic individuals. Decreased nitric oxide (NO) bioavailability contributes to diabetoporosis. This review summarizes the potential role of nitrate as a NO donor in preventing and treating diabetic osteoporosis. Evidence suggests that organic and inorganic nitrates have anti-osteoporotic effects in animal models of osteoporosis, as demonstrated by increasing bone mineral density (BMD, 3-42%) and bone weight (6-160%). Observational human studies indicate a lower fracture risk (6-17%) and a higher BMD (3-5%) following organic nitrate administration. Similar protective effects (7-74% reduction in fracture risk and 8-84% increase in BMD) have been observed with nitrate-rich diets. Randomized controlled trials have also shown that nitrate increases circulating bone formation markers; however, no effect on fracture risk has been reported, and increased BMD (8.8%) was reported only in one study. Nitrate converts to nitrite and then to NO (exogenous NO), increasing NO bioavailability in bone. In addition, nitrate increases the expression of endothelial NO synthase (eNOS), thereby increasing the endogenous NO in bone. Nitrate-derived NO promotes bone formation and reduces bone resorption via the NO/cyclic guanosine monophosphate (cGMP)/protein kinase G (PKG) signaling pathway. In addition to increasing NO availability, nitrate may enhance plasma insulin levels, reduce hyperglycemia, and improve insulin resistance in diabetes, further contributing to nitrates’ anti-osteoporotic effects in diabetic bone. In conclusion, NO-based interventions such as nitrate may have a potential role in preventing and treating diabetoporosis.

## 1 Introduction

Approximately 537 million people (10.5% of the global population) had diabetes in 2021, and this number is projected to rise to 783 million (12.2%) by 2045 (1). A meta-analysis of observational studies from 2001 to 2020 (103,334,579 subjects aged 15-105 years in all continents) indicates that the prevalence of osteoporosis is about 23% in women and 12% in men (2). The prevalence of osteoporosis is higher by ~28% (~8-54%) in women and men with diabetes (3). In addition, individuals with type 1 diabetes (T1D) face a 6-7 times higher risk of osteoporotic fractures compared to normal subjects, whereas those with type 2 diabetes (T2D) have an increased risk ranging from 38% to 70% (4–7). Moreover, hip fractures occur 10-15 years earlier in diabetic patients (8), increasing the risk of all-cause mortality by 24% (9) [28% in men and 57% in women (10)] over the following 1-5 years (9–11). Osteoporosis results from a diabetes-related decline in bone quality and quantity (12), often called diabetoporosis (13, 14).

Diabetic patients with osteoporosis are treated with a combination of antidiabetic and anti-osteoporotic medications. The effect of primary antidiabetic drugs, such as metformin and insulin, on fracture risk is inconsistent (15). Meta-analyses of randomized controlled trials (RCTs) and observational studies suggest that these medications have a neutral effect on fracture risk (9); however, some reports indicate an increased risk of fractures (16, 17). On the other hand, there are currently no RCTs evaluating the metabolic effects of anti-osteoporosis medications in diabetic patients. *Post hoc* analyses of trial data indicate that first-line anti-osteoporotic treatments like bisphosphonates may have a neutral or unfavorable impact on metabolic parameters, including fasting glucose and insulin resistance (18–21). In addition, current anti-osteoporotic treatments are limited by cost, side effects, and efficacy, warranting new strategies for managing diabetoporosis.

Decreased nitric oxide (NO) bioavailability contributes to diabetoporosis (22). In patients with T1D (22) and T2D (23), decreased endothelial NO synthase (eNOS) and increased inducible NOS (iNOS) activity have been observed in bone cells. eNOS-derived NO facilitates bone formation, reduces the risk of osteoporotic fractures, enhances bone healing, and inhibits bone resorption, whereas iNOS-derived NO hinders bone formation and promotes resorption (24–26). The lack of NO bioavailability in diabetic bones is linked to disruptions in specific signaling pathways, suggesting that targeting these pathways might improve bone health. Studies conducted in ovariectomized rats (27) and postmenopausal women (28) show that NO boosting through organic and inorganic nitrates can protect against osteoporosis. Furthermore, inorganic nitrate, particularly in food-derived sources, may indirectly ameliorate hyperglycemia and insulin resistance (29), improving bone quality. The protective effect of NO against osteoporosis in T1D (22) and T2D (23) has been previously reviewed. This paper focuses on the potential role of nitrate (organic and inorganic) in preventing and treating osteoporosis in patients with T1D and T2D.

## 2 Evidence of anti-osteoporotic effect of nitrate in animal studies

Animal studies have addressed the anti-osteoporotic effects of both organic and inorganic nitrates. Specifically, nitroglycerin (NG), an organic nitrate, improves BMD, bone weight, and bone quality. Both organic and inorganic nitrates may play a beneficial role in mitigating osteoporosis in rat models; however, further research is needed to evaluate the effectiveness of inorganic nitrates.

**Table 1** summarizes the anti-osteoporotic effects of organic nitrate [NG as single dose (30–34) or multiple doses (35, 36) for 4-12 weeks] and inorganic nitrate (sodium nitrate in drinking water for 3 and 36 weeks) in animal models of osteoporosis, viz., ovariectomy-induced osteoporosis (27, 31–34, 36, 37) and corticosteroid-induced osteoporosis (31, 35). All studies were conducted in rats (aged 12-36 weeks), as recommended by Food and Drug Administration guidelines for induction of animal models of osteoporosis (38) and discussed in our previous report (39). In addition, all studies were conducted in female rats except one study (31) that assessed corticosteroid-induced osteoporosis in male rats. NG has been used as dermal ointment except in one study that used it by oral gavage (35). Regions of interest assessed following NG administration include the femur (30–36), tibia (31, 34), and lumbar spine (33, 35), as these are the main fracture sites in humans and are clinically relevant (39). In the case of sodium nitrate administration, only the tibia has been assessed (27, 37).

**Table 1:** Evidence of the anti-osteoporotic effect of organic and inorganic nitrate in rats.

Study	Year	Sex	Age (weeks)	Osteoporosis model	Nitrate type	Dose^a^	Route	Duration (weeks)	Bone type	BMD	Bone weight	Bone quality^b^	Bone turnover markers	
													Serum	Urine
Wimalawansa, et al. (30)	1996	F	12	OVX	NG	0.2	Dermal ointment	6	Femur	↑ (10%)	↑ (67%)	-	-	-
Wimalawansa, et al. (31)	1997	M	32	CORT.	NG	0.4	Dermal ointment	6	Distal femur	-	↑ (7.8%)	↑ TBV (30%), cortical areas (50%)	↑ OC (54%)	-
									Proximal tibia	↑ (9.5%)				
Wimalawansa, et al. (32)	2000	F	36	OVX	NG	0.2	Dermal ointment	6	Femur	↑ (11.7%)	↑ (160%)	-	↔ OC	↓ DPD (292%)
Wimalawansa, et al. (33)	2000	F	28	OVX	NG	0.2	Dermal ointment	10	Lumbar spine	↑ (6.2%)	-	↑ TBV (9%)^c^	↑ OC (29%) ↑ ALP (32%)	↓ DPD (196%)
									Femur	-	↑ (71%)			
Hukkanen, et al. (34)	2003	F	12	OVX	NG	1	Dermal ointment	4	Proximal femur	↑ (12.5%)	-	-	↔ ALP	↔ DPD
									Distal femur	↑ (29.6%)				
									Proximal tibia	↑ (41.5%)				
Li, et al. (35)	2007	F	12	CORT.	NG	0.2, 0.4 1.0	Oral gavage	12	Lumbar spine	↑(10.3%)^d^	-	-	↑ OC (29%) ↔ALP ↓ TRAP (12.5%)	-
									Femur	↑ (11.2%)^d^				
Hao, et al. (36)	2005	F	12	OVX	NG	0.2, 0.4, 2.0	Dermal ointment	12	Femur	↑ (2.6%)^d^	↑ (6%)	↑ Calcium content	-	-
Conley, et al. (37)	2017	F	24	OVX	SN	140, 1400	Drinking water	3	Tibia	↔		↔ Cortical areas and thickness	↔ OC ↔ CTX	-
Yousefzadeh, et al. (27)	2022	F	24	OVX	SN	100	Drinking water	36	Proximal tibia	-	-	↑ TBV (42%) ↑ Tb.N. (61%) ↑ Tb.Th (12%) ↓ Tb.Sp.(15%)	-	-

ALP, alkaline phosphatase (bone formation marker); BMD, bone mineral density; BV/TV, bone volume/tissue volume; CORT., corticosteroid; CTX, C-terminal telopeptide of type I collagen (bone resorption marker); DPD, deoxypyridinolines (bone resorption marker); F, female; M, male; NG, nitroglycerin; OC, osteocalcin (bone formation marker); OVX, ovariectomy; TBV, trabecular bone volume; Tb.N, trabecular number; Tb.Th, trabecular thickness. TRAP, tartrate-resistant acid phosphatase (bone resorption marker).

^a^mg/kg/day for nitroglycerine (NG) and mg/L for sodium nitrate (SN); ^b^cortical and trabecular bone quality; ^c^higher frequency of administration (twice or three times once) has less effects rather than once daily; ^d^NG at a dose of 1 and 2 mg/kg/day had no effect. ↑, increase; ↓, decrease; ↔, no change.

Outcome variables that have been assessed included bone mineral density (BMD) (30–37), bone weight (30–33, 36), bone quality (27, 31, 33, 36, 37), and circulating bone turnover markers (31–37). Results show that organic nitrate has anti-osteoporotic effects (30–36), as documented by increasing BMD (2.6-41.5%) and bone weight (6-160%), improving bone quality, and affecting circulating and urine bone-related markers in favor of bone formation. In addition, it has been observed that a higher frequency of administration (twice or three times per day) rather than once daily (33), as well as a higher dose of NG compared to lower or moderate doses (35), provide less protection against osteoporosis in rats. Studies addressing the effect of inorganic nitrate on osteoporosis are scant (27, 37), with results indicating that in ovariectomy-induced osteoporosis in female rats, short-term nitrate administration (3 (37) and 4 (27) weeks) does not affect bone quality, but long-term administration [13 and 36 weeks (27)] does.

## 3 Evidence of the anti-osteoporotic effect of nitrate in human studies

### 3.1 Human studies: organic nitrates

**Table 2** summarizes evidence obtained from human studies on the association between organic nitrate and fracture risk, BMD, and bone turnover markers. Two case-control studies conducted in Denmark (45) and the Netherlands (46) compared fracture risk between men and women who consumed NG, isosorbide mononitrate (ISMN), and isosorbide dinitrate (ISDN) compared controls. Results indicate that nitrate consumption is associated with lower fracture risk (6-17%) (45, 46). In addition, a prospective cohort study (with a follow-up mean of 3.5 years) conducted in postmenopausal women in the United States indicated that intermittent nitrate use (NG, ISMN, and ISDN) is associated with higher BMD in the hip (~2.6%) and heel (~5.3%) (44). Five RCTs (28, 40–43) were conducted in postmenopausal women [except one study in young oophorectomized women aged 36-45 years (40)] to find the effect of NG as dermal ointment (40, 42) and transdermal patch (43, 45, 46) as well as ISMN as oral tablets (41) and transdermal patch (43) on BMD and bone turnover markers. Regions of interest were lumbar spine (28, 40, 42, 43), hip (40, 42, 43), and femoral neck (28, 42). None of the five RCTs addressed the effect on fracture risk (28, 40–43). In addition, only one study reported that ISMN (40 mg/kg once daily for 36 weeks) increases BMD in Indian postmenopausal women by 8.8% (28); the others observed no effect on BMD (40–43). Except for two studies (28, 42), all other four RCTs reported changes in bone turnover markers in favor of bone formation, as indicated by an increase in serum bone formation markers [i.e., bone-specific alkaline phosphatase (BSALP), by 15-25%] and decrease in urine bone resorption marker [i.e., N-telopeptide (NTx) by 32-40%]. In this line, a meta-analysis of RCTs that assessed the effect of antiresorptive agents in postmenopausal women indicates that a 40% reduction in bone resorption markers is associated with a 30% decrease in risk fracture (47).

**Table 2:** Evidence of the anti-osteoporotic effect of organic nitrate in humans.

Study	Year	National	Condition	n	Age (years)	Nitrate type	Dose (mg/day)	Route	Frequency of use (daily)	Duration (weeks)	Study design	Bone type	Fracture risk	BMD	Bone turnover markers	
															Serum	Urine
Wimalawansa, et al. (40)	2000	United States	Oophorectomized women	16	36–45	NG	15	Dermal ointment	Once	52	RCT	Hip, lumbar spine	-	↔	↓OC (53%), ↑ BSALP (27%)	↓ NTx (25- 40%)
Jamal, et al. (41)	2004	Canada	Postmenopausal women	144	57–60	ISMN	2.5, 5, 20	Orally tab	Once	13	RCT	-	-	-	↑ BSALP (16%)	↓ NTx (36%)
Wimalawansa, et al. (42)	2009	United States	Postmenopausal women	186	40-65	NG	22.5	Dermal ointment	Once	156	RCT	Hip, lumbar spine, femoral neck	-	↔	-	-
Duhan, et al. (28)	2012	India	Postmenopausal women	90	59.4	ISMN	40	Orally tab	Once	39	RCT	Lumbar spine	–	↑ (8.8%)	–	–
Bolland, et al. (43)	2020	New Zealand	Postmenopausal women	240	≥55	NG	25, 50	Transdermal patch	Once	52	RCT	Hip, lumbar spine, femoral neck	–	↔	↔	–
						ISMN	30, 60									
Jamal, et al. (44)^a^	1998	United States	Postmenopausal women	6201	≥65	NG, ISMN, ISDN	15	–	Once	183	Prospective cohort	Hip	↔	↑ (2.6%)	–	↔
												Heel	↔	↑ (5.3%)		
Rejnmark, et al. (45)^b^	2006	Denmark	Women and men	498617	17-68	NG	15	Transdermal patch	Once	261	Case-control	Hip	↓ (12%)	–	–	–
												Forearm	↓ (6%)			
						ISMN, ISDN^d^	30	Orally tab				Lumbar spine	↓ (7%)			
Pouwels, et al. (46)^c^	2012	Netherlands	Women and men	33104	18-90	NG	15	Transdermal patch	Once	574	Case-control	Hip	↓ (7-17%)	–	–	–
						ISMN, ISDN	30	Orally tab								

BMD, bone mineral density; BSALP, serum bone-specific alkaline phosphatase (bone formation marker); CTX, C-terminal telopeptide of type I collagen (bone resorption marker); ISMN, isosorbide mononitrate; ISDN, isosorbide dinitrate; NG, nitroglycerin; NTx, N-telopeptide (bone resorption marker); OC, osteocalcin; RCT, randomized, clinical trial.

^a^NG was used in two forms: Once per day and intermittent; lower frequency exposure (intermittent) had more beneficial effects; ^b^NG was used in three forms: Once, twice, or thrice per day; decreased risk of forearm fracture was reported only in women and decreased risk of vertebral fractures was reported only in men; ^c^NG was used in two forms: as-needed and maintenance; as-needed has more effect than maintenance; ^d^among users of ISMN and ISDN, decreased fracture risk was only marginally significant. ↑, increase; ↓, decrease; ↔, no change.

Of note, higher doses of NG (22.5 (42), 25 (43), and 50 (43) mg/day) provide less protection against osteoporosis in humans compared to 15 mg/day (40, 44–46). It has been reported that NG has a narrow therapeutic window for osteoporosis treatment, with optimal dosages of around 15 mg daily (48, 49). Deviations from this dosage, either too low or too high, may result in a lack of efficacy (42, 50, 51). Therefore, the reported ineffectiveness of NG in some instances may be associated with higher doses of NG (22.5 (42), 25 (43), and 50 (43) mg/day). In addition, a case-control study reported that using NG as a fast-acting nitrate in adult women and men had a more significant impact on fracture risk than using slow-release nitrates, such as ISMN and ISDN (45).

### 3.2 Human studies: inorganic nitrates

According to meta-analyses of observational studies, the Mediterranean diet is associated with a reduced risk of fractures by 20% in the general population (52, 53). These beneficial effects are hypothesized to be attributable to this diet’s high content of calcium, potassium, polyphenols, and fiber (54, 55). Furthermore, the high levels of inorganic nitrate found in fruits and vegetables are involved in the mechanisms underlying the positive effects of these diets (56, 57). Nitrate-rich vegetables account for approximately 85% of dietary nitrate consumption in the human diet (56, 57). These vegetables have potential NO-boosting effects (58) as their nitrate is converted to NO via the nitrate-nitrite-NO pathway (59), which may exert NO-like effects on bone. The protective effects of the Diet to Stop Hypertension (DASH) and Mediterranean diets against cardiovascular disease (60) and T2D (61) are at least partly attributed to the high levels of nitrates (147–1222 mg/day) derived from these nitrate-rich diets. This contrasts with the Western-style diet, which is considered a low-nitrate diet (75 mg/day) (62). For further support, meta-analyses of RCTs indicate that inorganic nitrate and beetroot juice supplementation produce similar blood pressure-lowering effects, while the presence of other bioactive compounds in beetroot juice, such as vitamin C, magnesium, and flavonoids, has minimal additive effects (63).

**Table 3** summarizes evidence obtained from human studies on the association between inorganic nitrate and fracture risk, BMD, and bone turnover markers. Five cross-sectional studies (84–88) were conducted in the UK (84), Iran (85), China (86), United States (87), and Taiwan (88) in women and men (84, 87), postmenopausal osteopenic women (85), and postmenopausal women (86, 88) who consumed fruit and vegetables. Results indicate that fruit and vegetable consumption as a source of nitrate is associated with lower fracture risk (~9%) (88) and higher BMD (84, 86, 87). Three case-control studies (81–83) conducted in China (81, 83) and the Netherlands (82) indicate that consumption of fruit and vegetables decreases fracture risk (81–83) and increases BMD (82). In addition, 11 prospective cohort studies (70–80) (with a mean follow-up of 146-740 weeks) have been conducted in Women and men (70, 71, 74–79), postmenopausal women (72, 73, 80) and men (73, 80) in the United States (70, 80), Sweden (79), Singapore (78), Europe (74, 77), France (75, 76), UK (71), Scotland (72), and Canada (73) to find association between inorganic nitrate consumption and bone-related parameters. Results indicated that nitrate use [fruit and vegetables (70, 71, 73, 76, 78–80), vegetables (74), and Mediterranean diet (75, 77)] is associated with lower fracture risk (74, 77–79) and higher BMD (70, 72, 73) in the hip (70, 71, 75–80), femoral neck (72, 73), lumbar spine (72), and wrist (75, 76). To summarize, results of observational studies indicate that higher inorganic nitrate consumption is associated with higher BMD (~1.3-8.8%) (70, 72, 73, 82, 84, 86, 87) (in case-control (82), cross-sectional (84, 86, 87) and prospective cohort (70, 72, 73) studies) as well as lower fracture risk (~ 7-74%) (in case-control studies (81–83) and prospective cohort (73, 74, 76–79) studies). In this line, a meta-analysis of cohort studies from Europe and the United States indicates that men and women consuming ≤1 serving per day of fruits and vegetables had a 39% higher risk of hip fractures compared to those consuming >3 and ≤5 serving (89).

**Table 3:** Evidence of anti-osteoporotic effect of inorganic nitrate in human studies.

Study	Year	National	Population	Study design	N	Age (years)	Nitrate type	Amount (g/day)	Duration (weeks)	Regions of interest	Fracture risk	BMD	Circulating bone turnover markers
Lin, et al. (64)	2003	France	women and men	RCT	186	23–76	DASH diet	≥ 400	4.3	–	–	–	↓ OC (8–11%), ↓ CTX (16–18%)
Macdonald, et al. (65)	2008	United States	Women and men	RCT	276	55-65	F&V	300	104	Hip	–	↔	↑ PINP (3%), ↔ CTX
McTiernan, et al. (66)	2009	United States	Postmenopausal women	RCT	48835	50-79	F&V	≥ 400	423	Hip	↔	↔	–
Ebrahimof, et al. (67)	2009	Iran	Postmenopausal osteopenic women	RCT	45	50-60	F&V	≥ 480	13	–	–	–	↓ OC (15%), ↓ CTX (4%)
Neville, et al. (68)	2014	United States	Women and men	RCT	83	65-85	F&V	≥ 160-400	17	–	–	–	↔ OC, ↔ CTX
Gunn, et al. (69)	2015	New Zealand	Postmenopausal women	RCT	138	50-70	F&V	≥ 720	13	–	–	–	↔ CTX, ↓ (8%) P1NP
Tucker, et al. (70)	1999	United States	Women and men	Prospective cohort	1164	69-97	F&V	–	209	Hip	–	↑	–
Kaptoge, et al. (71)	2003	UK	Women and men	Prospective cohort	944	65-74	F&V	–	146	Hip	–	↔	–
Macdonald, et al. (72)	2004	Scotland	Premenopausal women	Prospective cohort	891	45-55	F&V	–	261-365	Femoral neck, lumbar spine	–	↑	–
Langsetmo, et al. (73)	2011	Canada	Postmenopausal women and men	Prospective cohort	5188	≥ 50	F&V	–	349	Femoral neck	↓ (14-17%)	↑	–
Benetou, et al. (74)	2011	Europe	Women and men	Prospective cohort	29122	60-86	V	–	417	Hip	↓ (7%)	–	–
Feart, et al. (75)	2013	France	Women and men	Prospective cohort	1482	≥ 67	MD	–	417	Hip, wrist	↔	–	–
Samieri, et al. (76)	2013	France	Women and men	Prospective cohort	1482	68-95	F&V	–	417	Hip	↓ (14%)	–	–
										Wrist	↓ (19%)		
Benetou, et al. (77)	2013	Europe	Women and men	Prospective cohort	188795	48.6 ± 10.8^a^	MD	–	432	Hip	↓ (7%)	–	–
Dai Z, et al. (78)	2014	Singapore	Women and men	Prospective cohort	63257	45-74	F&V	–	470	Hip	↓ (26-39%)		–
Byberg, et al. (79)	2015	Sweden	Women and men	Prospective cohort	75591	45-83	F&V	–	516	Hip	↓		–
Fung, et al. (80)	2015	United States	Postmenopausal women and elderly men	Prospective cohort	109991	≥ 50	F&V	–	740	Hip	↔		–
Xie, et al. (81)	2013	China	Women and men	Case-control	1292	70.7 ± 6.8 ^a^	F&V	284-304	281	Hip	↓ (47-75%)	–	–
De Jonge, et al. (82)	2017	Netherlands	Women and men	Case-control	6331	≥ 55	F&V	NR	772	Hip	↓ (15%)	↑ (84%)	–
Xu, et al. (83)	2009	China	Postmenopausal women	Case-control	418	50-70	F&V	>370	–	Forearm	↓ (74%)	–	–
Prynne, et al. (84)	2006	UK	Women and men	Cross-sectional	520	16-83	F&V	>400	NA	Hip, lumbar spine, femoral neck	–	↑ (8%)	–
Ebrahimof et al. (85)	2006	Iran	Postmenopausal osteopenic women	Cross-sectional	48835	45-60	F&V	≥ 400	NA	Hip, lumbar spine	–	↔	↓ OC
Chen, et al. (86)	2006	China	Postmenopausal women	Cross-sectional	670	48-63	F&V	NR	NA	Hip, lumbar spine	–	↑	–
Zalloua, et al. (87)	2007	United States	Women and men	Cross-sectional	12055	25-64	F&V	≥ 250	NA	Hip	–	↑ (22-27%)	–
Lin, et al. (88)	2013	Taiwan	Postmenopausal women	Cross-sectional	1050	45-90	V	NR	NA	Any fracture	↓ (9%)	–	–

BMD, bone mineral density; CTX, C-terminal telopeptide of type I collagen; DASH, Dietary Approaches to Stop Hypertension; F&V, fruit and vegetable; MD, Mediterranean diet; NA, not applicable; OC, osteocalcin as a marker of bone formation; PINP, procollagen type I N-propeptide (a bone formation marker); RCT, randomized, double-blinded, controlled clinical trial.

^a^data are mean ± SD. ↑, increase; ↓, decrease; ↔, no change.

Six RCTs [duration range: 30 days (64) to 8.1 years (66)] were conducted in women and men (64, 65, 68), postmenopausal women (66, 69), and postmenopausal osteopenic women (66), to determine the effect of inorganic nitrate in the form of fruit and vegetables (65–69) [except one study with DASH diet (64)] on the fracture risk (66), BMD (65, 66) and circulating bone turnover markers (64, 65, 67–69). The anti-osteoporotic effect of inorganic nitrate has been assessed only in hip (65, 66). Results indicate that inorganic nitrate (300 and ≥ 400 g/day consumption of fruit and vegetables for 104 (65) and 423 (66) weeks) could not decrease the fracture risk (66) and BMD (65, 66). However, the consumption of inorganic nitrate for 4.3 (64), 13 (67), and 104 (65) weeks could decrease serum C-terminal telopeptide of type I collagen (CTX) levels by 4-18% and serum OC levels by 8-15% and increase procollagen type I N-propeptide (PINP) levels by 3-8%. A systematic review and meta-analysis of RCTs and cohort studies (13 studies) in men and women over 50 years of age conducted by Brondani et al. in 2019 reported an association between increasing fruit and vegetable intake by at least one serving per day and lower fracture risk (90).

**Table 3** shows that some RCTs indicate no effect of inorganic nitrate consumption on fracture risk, BMD, and serum bone turnover markers. Although participant characteristics may partly explain the lack of a significant effect of inorganic nitrate consumption on bone health outcomes, it seems that dosage inadequacy has a more critical role. Macdonald et al. (65) noted that more than a daily intake of 300 g of fruits and vegetables may be required to impact bone health significantly. Neville et al. (68) suggested that elderly participants (65-85 years) needed a higher dose (400 g/day) and longer treatment duration (over 16 weeks) of fruit and vegetable consumption to see meaningful effects on bone. Research suggests that at least 6.2 g of fresh fruit and vegetables per kg of body weight are needed to inhibit bone resorption. Considering each serving to be 80 g and a body weight of 70 kg, consuming five servings daily (400 g) is necessary to show an effect on bone resorption markers. According to a cohort study, men and women with no fruit or vegetable consumption had an 88% higher hip fracture rate than those consuming five servings daily (79); however, no additional benefits were observed for intakes exceeding five servings (720 g) (69).

### 3.3 Organic nitrates vs. inorganic nitrates

Organic nitrates are often poorly tolerated, with headache being their primary side effect; in an RCT, 21% of women discontinued the study during the 1-year follow-up because of headaches (43). Furthermore, the anti-osteoporotic effects of organic nitrates diminish with increased frequency (33) and duration (46) of administration. Inorganic nitrates have been suggested as suitable alternatives to organic nitrates (91). Inorganic nitrates have simple ionic structures, are produced endogenously, are present in the diet, and exhibit more prolonged effects without the limitation of tachyphylaxis (92, 93). Inorganic nitrates increase NO bioavailability following reduction to nitrite and then to NO (exogenous NO) and also enhance eNOS-derived NO (endogenous) in bone.

## 4 Bio-conversation of nitrate to nitrite and then to NO

At least two major pathways contribute to NO production in the human body (1): the *L*-arginine-NO-oxidative pathway in which NOS enzymes convert *L*-arginine to NO; (2) the nitrate-nitrite-NO reductive pathway in which the inorganic nitrate and nitrite are reduced to form NO (94).

In addition to the endogenous source (oxidation of NOS-derived NO), nitrate also has an exogenous source (diet, water, and environment) (57). Exogenous nitrate (1700 μmol/day), about 85% of which is derived from vegetables, is almost completely absorbed into circulation in the upper small intestine (duodenum and jejunum), with < 2% reaching the terminal ileum and excreted in feces. ~75% of ingested nitrate is excreted in the urine, while ~25% is transported into the salivary glands and concentrated in saliva (93–96). Oral bacteria utilize nitrate as an alternative electron acceptor during respiration, reducing the anion to nitrite. The saliva’s nitrite subsequently enters the stomach’s acidic environment, where it is reduced to NO and diffuses into the circulation. Considering that 25% of ingested nitrate is taken up from plasma by saliva and that 20% is converted to nitrite in the oral cavity, ~5% of ingested nitrate is converted to nitrite in the oral cavity (93–95). Assuming that all produced nitrite is reduced to NO in the stomach, the contribution of the nitrate-nitrite-NO pathway to overall NO production is estimated to be around 100 μmol/day, compared to the ~1000 μmol/day produced by the NOS-dependent *L*-arginine-NO pathway (57).

**Figure 1:**
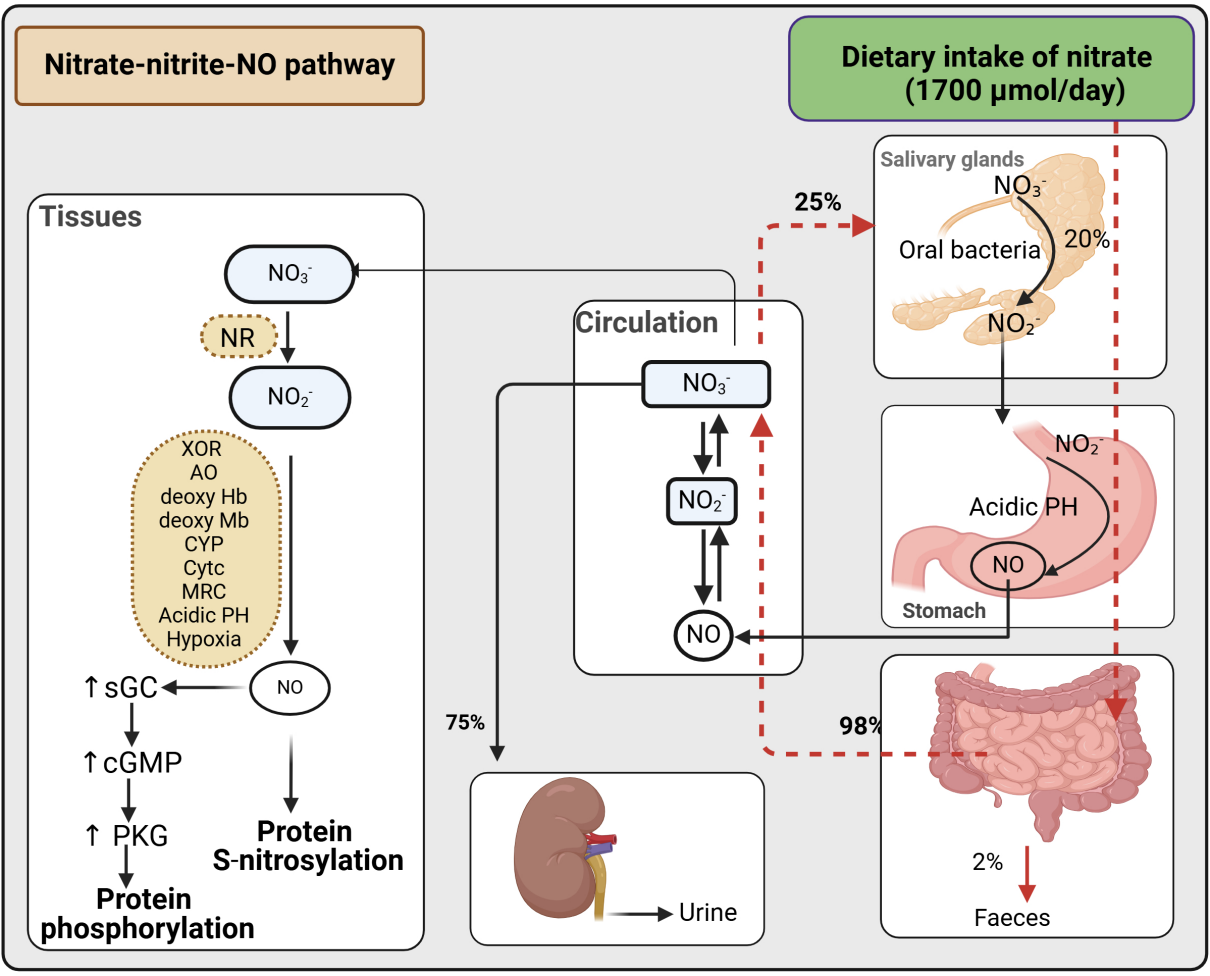
Bio-conversation of nitrate to nitrite and then to NO. cGMP, cyclic guanosine monophosphate; sGC, soluble guanylate cyclase; NO, nitric oxide; PKG, protein kinase G; NR, nitrate reductases; XOR, xanthine oxidoreductase; AO, aldehyde oxidase; deoxy HB, deoxygenated hemoglobin; deoxy MB, deoxygenated myoglobin; CYP, cytochrome P450; Cytc, cytochrome c; MRC, mitochondrial respiratory chain. Created with BioRender.com.

Nitrate reduction to nitrite and then to NO occurs in blood and tissues. Mammalian tissues express nitrate reductase and thus can reduce nitrate to nitrite under normoxic conditions (97). Nitrite reduction to NO can occur via enzymatic and non-enzymatic (i.e., spontaneously in an acidic environment or disproportionation) pathways (57, 96). Enzymes involved in the reduction of nitrite to NO include xanthine oxidoreductase, aldehyde oxidase, deoxygenated hemoglobin and myoglobin, cytochrome P450, cytochrome c, and the mitochondrial respiratory chain) (57, 96). Disproportioination is limited to the stomach and ischemic tissues because of low pKa of nitrite (~3.4) (98). The rate of NO production from nitrite disproportionation in normal tissues with a pH of 7.2 to 7.4 and nitrite concentrations of 10 to 50 μM is 0.05-1 pM/s (99), which is equivalent to about 6 μmol/day in a 70-kg individual. Tissues produce NO from nitrite under normoxia, increasing production in hypoxic conditions (97, 100). Under ischemic conditions, nitrite disproportionation can increase to 4-100 pM/s, but this is still only 5-10% of the maximum NOS-dependent NO production under physiological conditions (99). A decrease in pH enhances NO production from nitrite; in the presence of nitrite (20 μM), a one-unit decrease in pH from 7.0 to 6.0 increases NO generation by ~12 -13 times in liver and heart tissues (100). Thus, non-enzymatic reducing nitrite to NO is crucial in ischemic conditions in which both hypoxia and acidic pH are present. The combined inhibition of xanthine oxidase and aldehyde oxidase decreases NO generation from nitrite by more than 65-70%, indicating the significant contribution of these enzymes to nitrite reduction to NO, with only 15-20% remaining for non-enzymatic reduction of nitrite (100). See published reviews for more details about the nitrate-nitrite-NO reductive pathway (93–95) and the quantitative aspects of NO production (57) (**Figure 1**).

## 5 Possible mechanisms underlying protective effects of nitrate in diabetoporosis

T1D and T2D exhibit different pathophysiological characteristics; however, they share several features, including hyperglycemia, insulin resistance, and insulin deficiency, which negatively affect bone cells, contributing to the development of diabetoporosis (22, 23). The pathophysiology of diabetoporosis in T1D (22) and T2D (23) have been previously reviewed by us. In brief, both types of diabetes decrease osteoblast-related bone formation and increase osteoclast-related bone resorption. Additionally, reduced levels of transforming growth factor beta (TGF-β) and increased levels of sclerostin in osteocytes have been observed in both forms of diabetes that cause lower osteoblast and higher osteoclast differentiation. Furthermore, decreased bone blood flow, increased inflammation and oxidative stress, advanced glycation end products (AGEs), and bone adiposity contribute to the development of diabetoporosis (22, 23). Regarding gestational diabetes and osteoporosis, it has been reported that a history of gestational diabetes is associated with a higher risk of osteoporosis in postmenopausal women (101). Like T1D and T2D, increased AGEs and inflammation are involved in osteoporosis in subjects with a history of gestational diabetes (101). In addition, due to concerns about the possible adverse effects of nitrate use during pregnancy, including methemoglobinemia, changes in embryonic cells, malignant transformations, and thyroid disorders, the use of dietary nitrate as a common supplement during pregnancy is currently a long way from bench to bedside (102).

**Figure 2:**
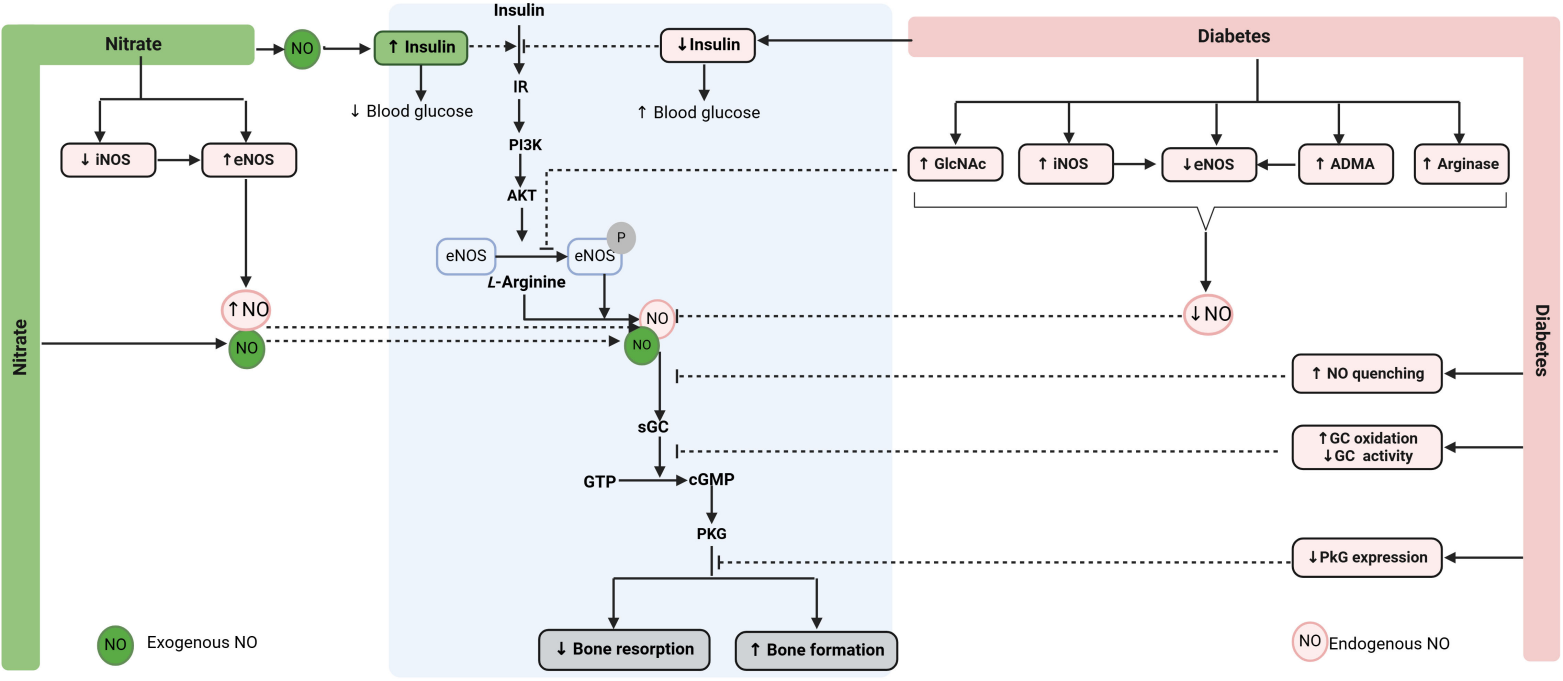
Proposed mechanisms by which nitrate can promote bone health in diabetoporosis. Nitrate is converted to NO (exogenous NO), increasing eNOS activity (endogenous NO), thereby restoring decreased NO bioavailability in diabetic bone. NO also increases circulating insulin and improves insulin resistance, indirectly promoting bone health in diabetes. ADMA, asymmetric dimethylarginine; cGMP, cyclic guanosine monophosphate; eNOS, endothelial nitric oxide synthase; GTP, guanosine triphosphate; GlcNAc, O-linked N-acetylglucosamine; IR, insulin receptor; iNOS, inducible NOS; sGC, soluble guanylate cyclase; NO, nitric oxide; PKG, protein kinase G. ↑, increase; ↓, decrease. Created with BioRender.com.

As shown in **Figure 2**, under normal conditions, insulin acts on insulin receptors (IR) and activates the phosphatidylinositol 3-kinase (PI3K)/Akt pathway in bone cells. Akt phosphorylates and activates eNOS, increasing endogenous NO production in bone cells. NO acts on its receptor (i.e., soluble guanylyl cyclase, sGC) and activates the sGC/cyclic guanosine monophosphate (cGMP)/protein kinase G (PKG) signaling pathway, the primary signaling pathway of NO action in the bone. sGC inhibitors block the effect of NO on bone (103), and restoring cGMP synthesis to normal levels in diabetic mice improves bone formation and decreases bone loss (104, 105). eNOS-derived NO increases osteoblast activity in bone as demonstrated by higher levels of ALP and osteocalcin (106, 107) and decreases osteoclast-mediated bone resorption as shown by reduced cathepsin K and collagenase levels in osteoclasts (108, 109). eNOS-derived NO also promotes osteoblast differentiation (110, 111) and inhibits osteoclast differentiation (110, 111). In addition, eNOS-derived NO represses adipogenesis by decreasing adipogenic transcription factors such as peroxisome proliferator-activated receptor γ (PPARγ) and lipoprotein lipase, thus decreasing adipogenesis in bone (112, 113). The ultimate effects of insulin-mediated eNOS activation in bone cells are increased osteoblast-mediated bone formation and decreased osteoclast-mediated bone resorption (114, 115). Supporting the favorable role of insulin on bone, reduced bone formation and increased bone resorption have been reported in rodents with insulin deficiency (115–118). In addition, insulin administration improves bone quality in diabetic rats (119–125).

Decreased NO bioavailability in diabetic bone is attributed to reduced availability of *L*-arginine (126), increased activity and expression of arginase [which converts *L*-arginine to urea and *L*-ornithine instead of NO] (126), increased levels of the asymmetrical dimethylarginine (ADMA) [which is an endogenous inhibitor of NOS] (127), decreased eNOS expression (105) and activity (128), increased eNOS uncoupling (105), increased expression and activity of iNOS (36), and increased *O*-linked *N*-acetylglucosamine (GlcNAc) [which inhibits eNOS phosphorylation] (129, 130). In addition, diabetes enhances AGE-mediated NO quenching (131), increases sGC oxidation that decreases its activity, and decreases PKG expression and activity in diabetic bone (105). These effects blunt PI3K/Akt/eNOS and NO/cGMP/PKG signaling pathways (105, 129), leading to osteoporosis.

Nitrate therapy can potentially restore decreased NO bioavailability in diabetoporosis as it is reduced to nitrite and then to NO (57). Nitrate also decreases iNOS expression and increases eNOS expression, thereby increasing endogenous eNOS-derived NO (132–134). Endogenous and exogenous NO can contribute to increased bone formation and decreased bone resorption via the NO/cGMP/PKG signaling pathway. In addition to increasing NO availability (57), nitrate increases plasma insulin, decreases hyperglycemia, and improves insulin resistance, as demonstrated in animal models of T2D (96, 135, 136). These effects can also contribute to nitrate-mediated health-promoting effects in diabetic bone.

## 6 Conclusion

Animal and human studies propose anti-osteoporotic effects for organic and inorganic nitrates. Animal studies (**Table 1**) indicate a 3-42% increase in BMD and a 6-160% increase in bone weight (femur, tibia, and lumbar spine) following the administration of NG and sodium nitrate in rat models of osteoporosis. Observational human studies indicate a 6-17% reduction in fracture risk and a 2.6-5.3% increase in BMD following organic nitrate administration (**Table 2**). Similar protective effects (a 7-74% reduction in fracture risk and an 8-84% increase in BMD) have been observed with nitrate-rich diets (**Table 3**). RCTs have shown increased circulating bone formation markers, and one study (**Table 2**) reported an 8.8% increase in BMD after the administration of ISMN in postmenopausal women; however, no effect on fracture risk has been reported. Inorganic nitrates may exert anti-osteoporotic effects in both T1D and T2D; however, further clinical trials in which BMD and fracture risk are considered the primary outcomes are needed to confirm the efficacy of inorganic nitrate in preventing and treating diabetoporosis.
